# MicroRNA-133b Alleviates Hypoxia Injury by Direct Targeting on NOD-Like Receptor Protein 3 in Rat H9c2 Cardiomyocyte

**DOI:** 10.1155/2019/8092461

**Published:** 2019-11-12

**Authors:** Yongmei Zhou, Hui Huang, Xiaolin Hou

**Affiliations:** Department of Cardiology, Sichuan Academy of Medical Sciences & Sichuan Provincial People's Hospital, Chengdu 610072, China

## Abstract

**Objective:**

MiR-133b was dysregulated in myocardial infarction. However, the role and mechanism of miR-133b in myocardial infarction remains unclear. This study was aimed to explore the role of miR-133b in H9c2 cell injury induced by hypoxia and to investigate the underlying molecular mechanism.

**Methods:**

Cell injury was assessed by cell viability, migration, invasion, and apoptosis assays. The expression of miR-133b and nucleotide-binding oligomerization domain-like receptor protein 3 (NLRP3) mRNA was determined by qRT-PCR. The levels of apoptosis-related proteins and NLRP3 were detected by western blotting.

**Results:**

Results showed that hypoxia significantly reduced cell viability, migration, and invasion, but increased apoptosis of H9c2 cells. Downregulation of miR-133b aggravated the cell injury induced by hypoxia. MiR-133b was directly targeted on NLRP3. Overexpression of NLRP3 significantly inhibited cell viability, migration, and invasion but induced cell apoptosis in H9c2 treated with hypoxia.

**Conclusions:**

Thus, miR-133b protects H9c2 against hypoxia injury via downregulation of NLRP3.

## 1. Introduction

Myocardial infarction (MI) is a common cardiovascular disease and is predicted to be a leading cause of future human death worldwide, which is caused by acute and persistent ischemia and hypoxia due to coronary artery occlusion [1, 2]. The severe and persistent myocardial ischemia and hypoxia induced cardiomyocyte death. After myocardial infarction, the left ventricular pump function will be further degraded and eventually lead to heart failure. In addition, abnormal discharge of myocardial cells after myocardial infarction will cause instability and fatal arrhythmia. Myocardial hypoxia is the basic clinical manifestation of coronary arteries in patients with acute myocardial infarction, and hypoxia-induced cell injury such as cell apoptosis is the major pathological change in infarcted regions [3]. Therefore, it is of great significance to investigate the molecular mechanism of myocardial hypoxia injury for developing novel treatment strategy for myocardial infarction.

MicroRNAs (miRNAs) are small single-stranded RNA with 18–25 ribonucleotides [4]. MiRNAs located on chromosomes transcribe the pri-miRNAs by the polymerase which is of hundreds to thousands of nucleotides in length. Pri-miRNAs are cleaved into miRNA precursors (pre-miRNAs) by the endonuclease. Pre-miRNAs are transported from the nucleus to the cytoplasm via transporters and ultimately form single-stranded miRNAs [5]. MiRNAs do not encode proteins, but can bind to the 3′ untranslated region of the target mRNA. Complete binding to miRNA can degrade the target mRNA and affect the transcription level of the target gene. If not complete binding, it can regulate the translation level by affecting the maturation, transport, and stability of the mRNA [6]. The main function of miRNAs is to regulate the basic processes of life, such as cell growth, proliferation, differentiation and cell apoptosis, aging, death, and so on [7, 8]. In recent years, it has been found that miRNAs can regulate the expression of genes related to cardiovascular diseases, such as miR-1, miR-21, miR-133, and miR-208, and are widely involved in pathological processes of cardiovascular disease such as myocardial fibrosis, cardiac hypertrophy, and arrhythmia [9, 10].

The miR-133 family includes miR-133a-1, miR-133a-2, and miR-133b. Among them, miR-133a is one of the most abundant miRNAs in the heart and plays an important regulatory role in cardiomyocyte differentiation and proliferation [11]. It was generally believed that miR-133b is expressed only in the muscle but not in the heart [12]. However, studies have shown that in cardiovascular disease tissues, the expression of miR-133a and miR-133b was both significantly changed [13, 14]. It was reported that miR-133a was decreased in MI, while miR-133b was slightly increased in the hearts of MI patients compared with that in the hearts of a healthy adult [14]. MiR-133a has been shown to have anticardiomyocyte apoptosis. Overexpression of miR-133a expression in myocardium significantly attenuates ischemia/reperfusion injury and improves cardiac function [14], possibly through negatively regulating the proapoptotic-related gene caspase-9 [15]. It was also reported that patients with occluded infarct-related artery had higher levels of miR-133b than patients with patent infarct-related coronary artery [13]. Although it was demonstrated that aberrant expression of miR-133b is involved in the regulation of cardiomyocyte apoptosis, the details in expression of miR-133b and role and mechanism in MI remains unclear. We hypothesized that hypoxia induced cardiac cell injury by downregulation of miR-133b and upregulation of its target gene. Herein, we predicted that the full-length sequence of nucleotide-binding oligomerization domain-like receptor protein 3 (NLRP3) is a direct target gene of miR-133b. The NLRP3 can sense intracellular danger signals such as ischemia during tissue injury [16]. It was demonstrated that NLRP3 is closely associated with the myocardial infarct size and the death of cardiomyocytes [17, 18]. NLRP3 aggravates MI injury in diabetic rats. Thus, we investigated the protective role of miR-133b in H9c2 cardiomyocytes against hypoxia injury and also explored the role of its target gene NLRP3 in the miR-133b action [19]. The miR-133b/NLRP3 pathway might be helpful for developing novel treatment strategy of myocardial infarction.

## 2. Materials and Methods

### 2.1. Cell Culture and Treatment

H9c2 cells were prepared from rat embryonic ventricular cardiomyocytes and cultured in DMEM supplementing with 10% fetal bovine serum, 100 *μ*g/ml streptomycin, and 100 U/ml penicillin at 37°C with 5% CO_2_. For hypoxia, cells were cultured in a hypoxic atmosphere with 94% N_2_, 5% CO_2_, and 1% O_2_ for 6 h. For transfection, miR-133b mimics (5′-GCUGGUCAAACGGAACCAAGU-3′) and inhibitor (5′-ACUUGGUUCCGUUUGACCAGC-3′) and their respective negative controls (NC) were purchased from Genepharma. The NLRP3 were constructed in pEX-2 plasmids (pEX-NLRP3, Genepharma). Transfection was performed using Lipofectamine 3000 (Invitrogen, USA) according to manufacturer's manual.

### 2.2. Rat MI Model

Ten-week-old male Wistar rats were maintained at 25°C, with a constant humidity of 55% and a cycle of 12 h of light and 12 h of darkness and had free access to food and tap water. MI was prepared by ligation of the left anterior-descending (LAD) artery. Rats were anesthetized with 10% chloralic hydras at 3 ml/kg. The ligation of the LAD procedure was described previously [20]. The sham operation group was without LAD occlusion. The MI was estimated on the basis of histological changes. The study was approved by the Animal Ethics Committee of Sichuan Academy of Medical Sciences and Sichuan Provincial People's Hospital.

### 2.3. Experimental Design

We used 9 rats in each groups, Sham and MI groups, respectively. At 24 h after the sham operation or coronary artery ligation, the cardiac tissues were collected for miR-133b detection. There are no dead rats in the sham-operated group. After myocardial infarction, no rats died after 24 h. Then, roles of miR-133b in cardiomyocytes were detected using miR-133 inhibitor or pEX NLRP3 overexpressing vector.

### 2.4. qRT-PCR

Total RNA was extracted from cells using Trizol reagent (Invitrogen, USA). For cDNA synthesis, the One Step SYBR® PrimeScript® PLUS RT-RNA PCR Kit (TaKaRa, China) was used. PCR was performed on ABI 7200 real-time PCR system (Applied Biosystems, USA) with SYBR Green PCR Kit (Takara, Japan). MiR-133 and U6 were performed using Taqman MicroRNA Reverse Transcription Kit and Taqman Universal Master Mix II with the TaqMan MicroRNA Assay. NLRP3 was normalized to GAPDH, and miR-133b was normalized to U6 by the 2−ΔΔCt method. The primer sequences were as follows: NLRP3 forward primer: 5′-ATT-ACCCGCCCGACAATAGG-3′, reverse primer: 5′-CATGAG-TCAGCTAGGCTAGAA-3′, miR-133b forward primer: 5′- CGGAATTCTATGTTGGTCCCTGGGCA-3′, and reverse primer: 5′-GCGGATCCCTTACAGACATACTGGTC-3′.

### 2.5. Cell Viability Assay

H9c2 cells (1 × 10^5^) were seeded in 60 mm culture dishes. After treatment, cell viability was analyzed by the TC20 Automated Cell Counter (BioRad, USA) based on Trypan blue excusion method [21].

### 2.6. Apoptosis Assay

After treatment, H9c2 cells were incubated with Annexin V-FITC-propidium iodide apoptosis kit (Beyotime, China) according to the manufacturer's manual. Cell apoptosis was detected on a CytoFLEX FCM Flow cytometer (Beckman Coulter, USA).

### 2.7. Cell Migration and Invasion

Transwell chambers (pore size of 8 *μ*m, Corning, USA) were used for evaluating migration and invasion capacities. Cells were seeded in the upper chamber, and medium with 10% fetal bovine serum was added in the lower chamber. After 24 h, the cells not migrated or invaded were removed by a cotton swab. The cells on the lower surface were fixed with 4% paraformaldehyde, stained with 0.1% crystal violet and counted. For invasion, the transwell chamber was precoated with Matrigel (BD, USA).

### 2.8. Luciferase Reporter Assays

Dual-Luciferase Reporter Assay System (Promega, USA) was performed according to the manufacturer's manual. The wide-type or mutant NLRP3 with miR-133b binding site was established, integrated into a pmir-GLO Dual-luciferase vector to form the vectors, and cotransfected with miR-133b into cells by Lipofectamine 3000. The luciferase activity was detected.

### 2.9. Western Blot

Proteins were extracted from cells using RIPA lysis buffer (Beyotime, China), and the concentrations were measured using BCA protein Assay (Beyotime, China). Proteins were separated using 10% SDS-PAGE and then transferred to PVDF membranes (Millipore, USA). After blocked in 5% nonfat milk and incubated with primary antibody against Bcl-2, Bax, pro-Caspaes-3, cleaved-Caspase-3, NLRP3, and GAPDH (Abcam, USA) overnight at 4°C, the membranes were incubated with HRP-conjugated secondary antibody at the room temperature for 1 hour. The bands were detected with the ECL detection system (Millipore, USA) and exposure to X-ray film.

### 2.10. Statistical Analysis

The data were repeated three independent times and presented as mean ± SD. Statistical analysis was performed with SPSS 20.0 (IBM, USA). Student's *t* test and one-way ANOVA analysis with Turkey's post hoc test were performed. *P* < 0.05 was considered as statistical significance.

## 3. Results

### 3.1. Hypoxia Induced Cell Injury of H9c2

**Figure 1:**
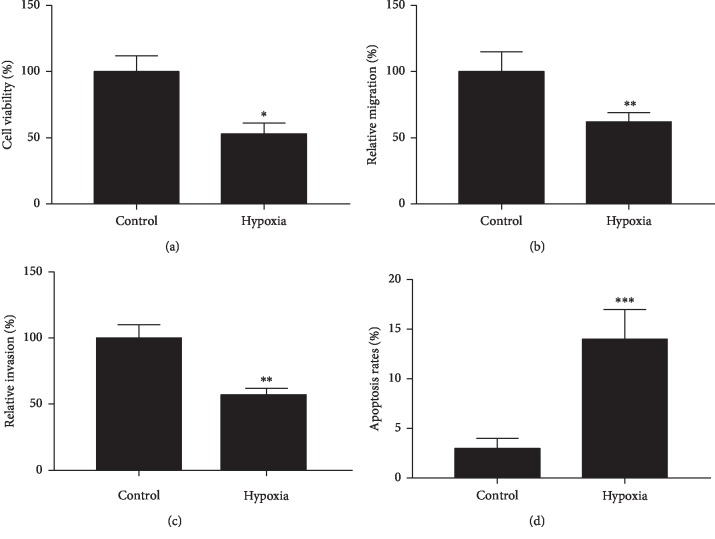
Effect of hypoxia on (a) Cell viability assay. (b) Migration assay. (c) Invasion assay. (d) Apoptosis assay. Error bars represent mean ± SD (*n* = 3). ^*∗*^*P* < 0.05; ^*∗∗*^*P* < 0.01; ^*∗∗∗*^*P* < 0.001.

**Figure 2:**
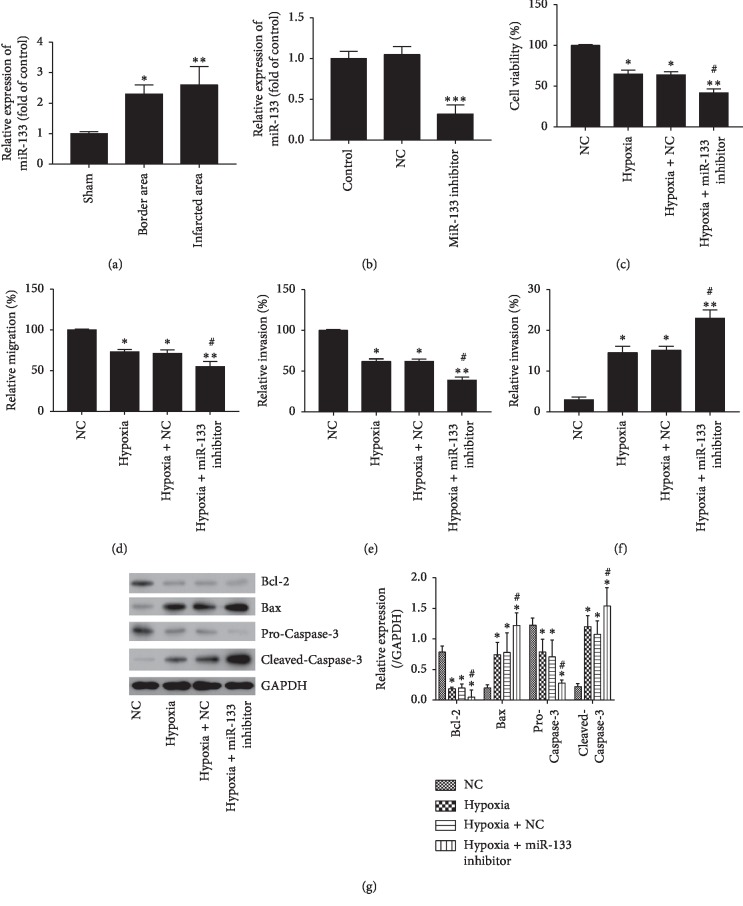
MiR-133b levels in MI and Sham rats and the change in cell damage by hypoxia in miR-133b or negative control (NC) inhibitor-transfected cells. (a) MiR-133b level in rat MI areas. (b) MiR-133b level in H9c2 cells after transfected with miR-133b or NC inhibitor. (c) Cell viability assay. (d) Migration assay. (e) Invasion assay. (f) Apoptosis assay. (g) The expression of apoptotic proteins Bcl-2, Bax, procaspase3, and cleaved-caspase3. Error bars represent mean ± SD (*n* = 3). ^*∗*^*P* < 0.05; ^*∗∗*^*P* < 0.01; ^*∗∗∗*^*P* < 0.001 vs. control; ^#^*P* < 0.01 vs. hypoxia.

Cell injury model induced by hypoxia was commonly used to reflect the conditions in MI and myocardial ischemia [22, 23]. After hypoxia treatment, the injury of H9c2 cells was confirmed firstly. As shown in Figure 1(a), cell viability of H9c2 cells was significantly reduced in hypoxia compared with control (*P* < 0.05). As shown in Figures 1(b) and 1(c), the migration and invasion of H9c2 cells in hypoxia were significantly inhibited compared with control (*P* < 0.01). However, the apoptosis rate of oxygen-deprived H9c2 was significantly increased (*P* < 0.001, Figure 1(d)). The expression of antiapoptotic protein Bcl-2 was downregulated, and proapoptotic proteins including Bax and cleaved-Caspase-3 were upregulated (Figure 2(g)). Altogether, these results suggested that hypoxia induced cell injury of H9c2.

### 3.2. MiR-133 Was Increased in MI and miR-133b Inhibitor Promoted Cell Injury Induced by Hypoxia

The levels of miR-133b in different areas of infarcted hearts in MI at early stage were determined. As shown in Figure 2(a), miR-133b was significantly upregulated in the border (*P* < 0.05) and infarcted areas (*P* < 0.01) at 24 h after MI. As shown in Figure 2(b), miR-133b inhibitor significantly downregulated the expression of miR-133b (*P* < 0.001). Knockdown of miR-133b significantly aggravated the inhibitory effects of hypoxia on cell viability, migration, and invasion of H9c2 cells (*P* < 0.05, Figures 2(c)–2(e)). Moreover, knockdown of miR-133b significantly enhanced the apoptosis rate of H9c2 cells induced by hypoxia (*P* < 0.05, Figure 2(f)). The proapoptotic proteins including Bax and cleaved-Caspase-3 in the hypoxia-treated H9c2 cells were further increased by the miR-133b inhibitor, and the antiapoptotic protein Bcl-2 was further downregulated (Figure 2(g)). Altogether, these results suggested that knockdown of miR-133b promotes the cell injury induced by hypoxia.

### 3.3. MiR-133 Targets on NLRP3

**Figure 3:**
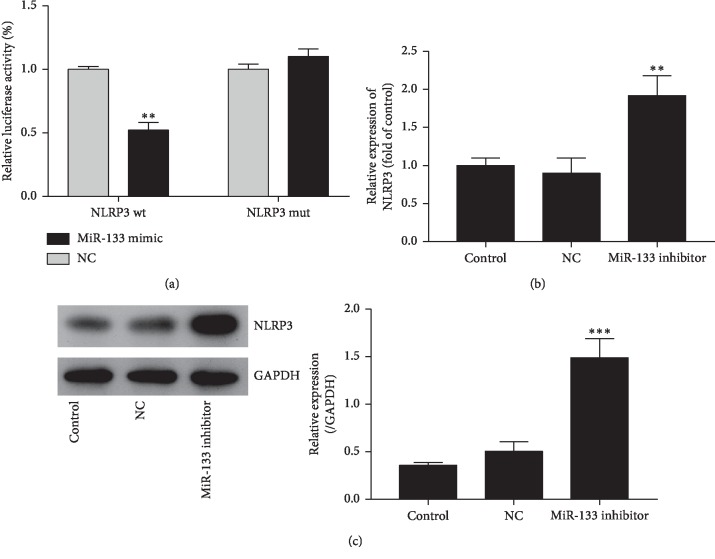
Dual luciferase reporter assay confirmed that NLRP3 is a direct target gene of miR-133b. (a) The luciferase activity of miR-133b was performed. The wide type (wt) or mutant (mut) NLRP3 with miR-133b binding site were integrated into a pmir-GLO dual-luciferase vector, and cotransfected with miR-133b or negative control (NC) mimics into cells. (b) The expression of NLRP3 mRNA in H9c2 cells. (c) The NLRP3 protein level in H9c2 cells. Error bars represent mean ± SD (*n* = 3). ^*∗∗*^*P* < 0.01, ^*∗∗∗*^*P* < 0.001 vs. control.

The luciferase activity was significantly decreased in cells that were cotransfected with miR-133b mimics and NLRP3 wt vector, but that of miR-133b mimics and NLRP3 mut vector-cotransfected cells were not significantly changed (Figure 3(a)), indicating that miR-133b might regulate the cell injury induced by hypoxia through NLRP3.

To confirm the role of miR-133b in regulating the NLRP3 expression, the levels of NLRP3 mRNA and protein in cells transfected with miR-133b inhibitor were detected. As shown in Figures 3(b) and 3(c), both NLRP3 mRNA and protein were significantly increased by knockdown of miR-133b.

### 3.4. NLRP3 Regulated the Cell Injury of H9c2 Induced by Hypoxia

**Figure 4:**
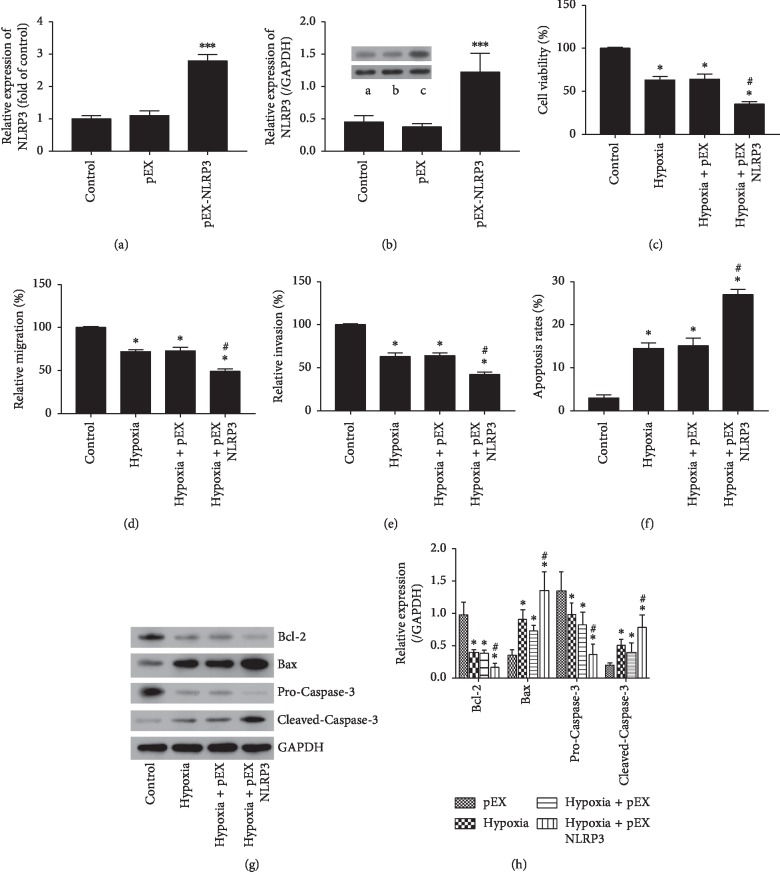
Regulated the cell damage of H9c2 by hypoxia in NLRP3-transfected or control cells. (a) Expression of NLRP3 mRNA. (b) Expression of NLRP3 protein (a: control; b: pEX; c: pEX-NLPR3). (c) Cell viability assay. (d) Cell migration assay. (e) Cell invasion assay. (f) Apoptosis assay. (g) Western blot detection of apoptotic proteins. (h) Quantification data. pEX: pEX-2 plasmids with empty vector; pEX-NLRP3: pEX-2 plasmids constructed NLRP3 vector. Error bars represent mean ± SD (*n* = 3). ^*∗*^*P* < 0.05; ^*∗∗∗*^*P* < 0.001 vs. control; ^#^*P* < 0.05 vs. hypoxia.

The role of NLRP3 in cell injury of H9c2 induced by hypoxia was detected. As shown in Figures 4(a) and 4(b), pEX-NLRP3 transfection significantly upregulated the NLRP3 expression (*P* < 0.001). Overexpression of NLRP3 significantly enhanced the inhibition of cell viability, migration, and invasion induced by hypoxia (*P* < 0.05, Figures 4(c)–4(e)). Moreover, overexpression of NLRP3 significantly promoted the apoptosis of H9c2 cells induced by hypoxia (*P* < 0.05, Figure 4(f)). Overexpression of NLRP3 upregulated the proapoptotic proteins and downregulated the antiapoptotic protein (Figure 4(g)). Altogether, overexpression of NLRP3 aggravated H9c2 cell injury induced by hypoxia.

## 4. Discussion

Myocardial infarction is a cardiovascular disease that seriously endangers human health and can cause poor prognosis such as heart failure. Hypoxia-ischemia injury of myocardial cells is a basic pathological change of myocardial infarction. The cell viability, migration, invasion, and apoptosis are important processes involved in myocardial injury and repair after myocardial infarction. This study indicated that the regulating mechanism of miR-133b on hypoxia induced cell injury in H9c2 cells by targeting on NLRP3.

MiRNAs can regulate cardiomyocyte apoptosis through its downstream target genes and signaling pathways [24], thus making miRNA as one of the targets for clinical treatment and prevention of cardiomyocyte apoptosis-related diseases [9]. MiR-133 family mainly includes miR-133a and miR-133b. Among them, miR-133a has been proved to be an important anticardiomyocyte apoptosis miRNA, which may inhibit cardiomyocyte apoptosis by targeting caspase-9 [14, 15]. MiR-133a can target cyclin D2 and regulate cardiomyocyte proliferation [25]. MiR-133a can inhibit myocardial fibrosis and improve electrophysiological effects in the models of transverse aortic constriction and isoproterenol-induced hypertrophy [26]. MiR-13a knockout can lead to ventricular septal defect and dilated cardiomyopathy in rats [25]. MiR-133b is highly homologous to miR-133a and may have similar biological functions. Unlike miR-133a, it was reported that miR-133b was slightly increased in hearts of MI patients compared with healthy adults [14]. In the patients with occluded infarct-related artery, it had higher cardiac miR-133b than the patent infarct-related coronary artery [14]. In this study, we found miR-133b was significantly increased in myocardial tissue in MI rats at early stage (24 h). Hypoxia induced H9c2 cell injury, which was enhanced by downregulation of miR-133b, indicating that miR-133b might play a vital role in antiapoptosis in cardiomyocytes. At early stage, the increase of miR-133b in an MI heart might contribute to protect the hypoxia injury. Maintaining or upregulating the expression of miR-133b may be beneficial to the survival of cardiomyocytes.

In this study, bioinformatics analysis and dual luciferase assay confirmed that NLRP3 is one of the direct target genes of miR-133b, and that miR-133b inhibitor can significantly upregulate the expression of NLRP3 mRNA and protein. Compared with normal oxygen conditions, hypoxia induced miR-133b expression and downregulated the NLRP3 in H9c2 cells (data not shown). Marchetti et al. [27] established a rat model of myocardial ischemia-reperfusion (ischemia for 30 min and reperfusion for 24 h) and found that inhibition of NLRP3 activity can reduce myocardial infarct size and cardiac troponin in rat serum. Inhibition of NLRP3 receptors by small interfering RNAs (siRNA) inhibited the death of cardiomyocytes and ultimately reducing myocardial remodeling after MI [17]. Consistent to these results, in this study, we found that overexpression of NLRP3 further inhibited cell viability, migration, and invasion under hypoxia. At the same time, the caspase-3 cascade was activated, which ultimately leads to apoptosis. In many diseases, NLRP3 is produced by triggering a risk signal to induce aseptic inflammation. The common upstream mechanisms of NLRP3 include intracellular *K* + efflux, lysosome rupture, and reactive oxygen species (ROS) [28]. Once the NLRP3 is activated, its PYD domain recruits ASC adapters and procaspase-1, which splits procaspase-1 into active caspase-1 [29]. How is caspase-3 activated by NLRP3 remains unknown; nevertheless, it is speculated that the inhibition of miR-133b may induce injury and apoptosis of cardiomyocytes through upregulating of NLRP3.

In conclusion, downregulation of miR-133b could induce injury and apoptosis of cardiomyocytes through upregulating NLRP3. These results provide novel role of miR-133b in MI and discover its target gene NLRP3, which could be used for MI treatment. In addition, the role of NLRP3 in MI might associate with inflammation, and this should be further studied in the future.

## Data Availability

The data used to support the findings of this study are available from the corresponding author upon request.

## Additional Points

*Highlights*. (i) Downregulation of miR-133b aggravated the cell injury induced by hypoxia. (ii) MiR-133b was directly targeted on NLRP3. (iii) Overexpression of NLRP3 alleviated hypoxia cell injury.
